# Clinical Evaluation of Oxidative Stress Biomarkers in Cirrhosis: Associations with Child–Pugh Class and Hepatic Encephalopathy

**DOI:** 10.3390/diagnostics15222853

**Published:** 2025-11-11

**Authors:** Vlad Pădureanu, Virginia Maria Rădulescu, Cristiana Gianina Moise, Marius Cristian Marinaș, Rodica Pădureanu, Denisa Marilena Săbiescu, Denisa Floriana Vasilica Pîrșcoveanu, Dragoș Forțofoiu, Lidia Boldeanu

**Affiliations:** 1 Department of Internal Medicine, University of Medicine and Pharmacy of Craiova, 200349 Craiova, Romania; vlad.padureanu@umfcv.ro (V.P.); rodica.padureanu@umfcv.ro (R.P.); denisa.sabiescu@umfcv.ro (D.M.S.); dragos.fortofoiu@umfcv.ro (D.F.); 2 Department of Medical Informatics and Biostatistics, University of Medicine and Pharmacy of Craiova, 200349 Craiova, Romania; virginia.radulescu@umfcv.ro; 3 Department of Cardiology, Craiova Emergency County Hospital, 200642 Craiova, Romania; 4 Department of Human Anatomy, University of Medicine and Pharmacy of Craiova, 200349 Craiova, Romania; 5 Department of Neurology, University of Medicine and Pharmacy of Craiova, 200349 Craiova, Romania; denisa.pirscoveanu@umfcv.ro; 6 Department of Microbiology, University of Medicine and Pharmacy of Craiova, 200349 Craiova, Romania; lidia.boldeanu@umfcv.ro

**Keywords:** biomarkers, liver cirrhosis, oxidative stress, inflammation, Child–Pugh score, hepatic encephalopathy

## Abstract

**Background/Objectives**: Oxidative stress contributes to the pathogenesis of cirrhosis, but its value as a clinical biomarker remains uncertain. **Methods**: We retrospectively analysed 90 patients with decompensated cirrhosis. Serum malondialdehyde (MDA) and 8-epi-prostaglandin F2α (8-iso-PGF2α) were measured at admission. Biomarker levels were compared between Child–Pugh classes B and C, across hepatic encephalopathy grades, and ascites severity, using Mann–Whitney, Kruskal–Wallis, and Spearman correlation tests. **Results**: Median MDA did not differ significantly between Child–Pugh classes B and C (2.67 [2.10–3.20] vs. 2.45 [1.98–3.05] μmol/L; *p* = 0.331), nor across ascites categories (*p* = 0.453). Similarly, 8-iso-PGF2α values did not vary between Child–Pugh classes (255.8 [220.0–310.0] vs. 250.1 [210.0–295.0] pg/mL; *p* = 0.784) or ascites groups (*p* = 0.828). Spearman analysis showed no significant correlations with albumin, INR, bilirubin, creatinine, or age, except for a non-significant trend with bilirubin (ρ = −0.18, *p* = 0.09). Importantly, MDA levels increased significantly across encephalopathy grades (*p* = 0.021), suggesting a link between systemic oxidative stress and neuropsychiatric impairment. **Conclusions**: In this clinical cohort, oxidative stress biomarkers did not provide discriminatory value for staging by Child–Pugh or ascites, but MDA was associated with encephalopathy severity. These findings highlight both the limitations and potential clinical relevance of oxidative stress markers in cirrhosis management.

## 1. Introduction

Oxidative stress, characterized by the imbalance between pro-oxidants and anti-oxidant capacity, significantly influences the progression of inflammatory, metabolic, and other chronic liver disease (CLD). Chronic liver injury may present as fibrosis, cholestasis, necrosis, and cirrhosis [1]. Liver cirrhosis represents the terminal phase of multiple forms of chronic liver disease, with fibrosis serving as its precursor. The global burden of liver disease is often underestimated, yet it continues to increase [2]. Alcohol consumption and chronic infections from the hepatitis B virus (HBV) and/or the hepatitis C virus (HCV) are primary causes of liver cirrhosis. In 2018, liver cirrhosis was identified as the 11th leading cause of mortality globally [3], with first-year mortality rates varying from 1% to 57% based on disease stage [1,4].

Pathological features universally observed in liver cirrhosis include hepatocyte degeneration and necrosis, the substitution of liver parenchyma with fibrotic tissue and regenerative nodules, and a decline in liver function. High exposure to alcohol induces structural and functional changes in the liver due to two interconnected processes: oxidative stress and inflammation [5]. Alcohol has the potential to elevate the production of reactive oxygen and nitrogen species (ROS, RNS), which can subsequently induce profibrogenic cytokines, trigger the release of various inflammatory markers, and promote collagen synthesis in the context of liver fibrosis progression [1,6]. Reactive oxygen species are molecules containing oxygen that are generated during standard metabolic processes. The organism possesses two systems for neutralizing the detrimental effects of endogenous reactive oxygen species: enzymatic and non-enzymatic antioxidants [7]. The liver typically maintains the equilibrium between internal antioxidants and ROS to effectively neutralize free radicals produced by viruses and various endogenous and exogenous substances processed by the organ. The oxidative to antioxidative balance can shift towards oxidative status under specific conditions, due to an increase in ROS production or a depletion of antioxidants. When the liver is subjected to persistent oxidative stress, the resultant free radical damage escalates, leading to inflammation and fibrosis [8].

Oxidative stress induces liver injury by altering key biological molecules, including deoxyribonucleic acid (DNA), proteins, and lipids [9]. Previous studies indicate that DNA and protein oxidation, along with lipid peroxidation products, modulate signaling pathways related to gene transcription, protein expression, apoptosis, and hepatic stellate cell activation, thereby contributing to the onset and progression of liver fibrosis [10,11]. Inflammation is a critical component of the immune response, characterized by the recruitment of inflammatory cells to combat diverse harmful stimuli.

The relationship between oxidative stress and inflammation in the development of liver disease has long captured researchers’ interest. Excessive inflammatory cells can generate increased levels of ROS and RNS, which in turn may enhance the expression of genes encoding proinflammatory cytokines. Oxidative stress and inflammation are closely linked, forming a vicious cycle that contributes to the progression of cirrhosis and ultimately hepatocellular carcinoma in liver diseases [12,13].

The present study aimed to evaluate the markers of oxidative stress, with the goal of developing new predictive tools for non-invasive paraclinical investigations of disease outcomes in patients with liver cirrhosis.

Among the most widely studied biomarkers of oxidative stress in chronic liver disease are malondialdehyde (MDA) and 8-epi-prostaglandin F2α (8-iso-PGF2α; F2-isoprostanes). MDA is a reactive end-product of lipid peroxidation that readily forms adducts with DNA and proteins, thereby modulating transcriptional regulation, apoptosis, and hepatic stellate cell activation. In patients with cirrhosis, serum MDA levels are consistently reported to be significantly elevated compared with healthy controls, and these elevations have been correlated with clinical severity indices such as Child–Pugh score, presence of ascites, and esophageal varices [14,15]. Furthermore, MDA has been associated with extrahepatic complications.

Nevertheless, the clinical interpretation of MDA is hampered by methodological limitations. The classical spectrophotometric TBARS assay often lacks specificity, as it can detect other aldehydes and reactive substances, thus overestimating true MDA concentrations. More advanced methods, such as high-performance liquid chromatography (HPLC) or gas chromatography–mass spectrometry (GC-MS), offer superior accuracy and reproducibility [16,17]. When measured by these robust approaches, MDA has shown strong associations with portal hypertension and liver decompensation, reinforcing its potential as a prognostic biomarker in cirrhosis.

In contrast, 8-iso-PGF2α is considered the gold standard marker of lipid peroxidation in vivo, due to its chemical stability, specificity for free-radical-mediated oxidation of arachidonic acid, and reliable quantification in both plasma and urine [18,19,20]. Elevated concentrations of 8-iso-PGF2α have been documented in alcoholic-related liver disease (ARDL), metabolic dysfunction-associated steatotic liver disease (MASLD), and cirrhosis. Importantly, urinary excretion of 8-iso-PGF2α decreases following alcohol abstinence, indicating its responsiveness to dynamic changes in oxidative stress [19,20]. Additionally, 8-iso-PGF2α is mechanistically linked to the activation of NADPH oxidase (particularly NOX2), a major enzymatic source of reactive oxygen species in immune and vascular cells [21]. Experimental studies have demonstrated that isoprostanes can stimulate collagen synthesis in activated hepatic stellate cells, directly contributing to fibrogenesis [22].

Analytically, GC-MS or LC-MS/MS are regarded as reference techniques for accurate measurement of 8-iso-PGF2α, while immunoassays may be used in clinical or epidemiological settings but require careful validation. Notably, urinary measurements are preferable, as they are less prone to artifactual generation ex vivo and provide a more reliable index of systemic oxidative stress [18,20,23].

Taken together, the combined assessment of MDA and 8-iso-PGF2α offers complementary insights: MDA reflects the systemic oxidative burden and correlates with disease severity, while 8-iso-PGF2α provides a dynamic and mechanistically relevant index of lipid peroxidation. Integrating both biomarkers may improve non-invasive risk stratification and monitoring of patients with liver cirrhosis.

Despite the widespread use of clinical scores such as Child–Pugh and MELD, there remains a clinical need for simple serum biomarkers that can support risk stratification and prediction of complications in cirrhotic patients. Oxidative stress markers, including malondialdehyde (MDA) and 8-epi-prostaglandin F2α (8-iso-PGF2α), have been proposed as potential tools, yet their clinical applicability is still uncertain due to heterogeneous results. The present study aimed to evaluate the clinical value of these biomarkers in a cohort of patients with decompensated cirrhosis, by analysing their associations with Child–Pugh class, ascites, and hepatic encephalopathy. We further explored whether integrating age with MDA levels could provide an additional tool for identifying patients at higher risk of severe disease.

Clinical relevance. Beyond their pathobiological interest, serum oxidative stress biomarkers may inform risk stratification and monitoring in cirrhosis. For example, 8-iso-PGF2α reflects ongoing lipid peroxidation and has shown dynamic responsiveness to changes such as alcohol abstinence, while MDA captures the systemic oxidative burden that plausibly contributes to complications like hepatic encephalopathy. Although these markers do not replace established scores (Child–Pugh, MELD-Na), integrating them with clinical and biochemical parameters could help flag patients at risk of worsening neurocognitive status or renal–vascular instability, thereby supporting individualized follow-up and adjunctive therapy evaluation. Accordingly, our primary objective was exploratory: to assess whether serum MDA and 8-iso-PGF2α differ across clinically defined severity strata (Child–Pugh class, West Haven HE grade, and International Ascites Club ascites grade) and to examine their simple associations with routine laboratory measures. A secondary, hypothesis-generating analysis evaluated a minimal composite (Age + MDA, median-based) purely as a signal-finding step; no clinical prediction model was proposed or validated.

## 2. Materials and Methods

### 2.1. Study Design and Analyzed Population

Between October 2024 and March 2025, we screened all consecutive admissions for decompensated liver cirrhosis at the 2nd Medical Clinic of the Craiova County Emergency Hospital. This was a retrospective cohort: patients were identified from medical records, and serum aliquots collected during routine hospitalization had been stored at −80 °C in the institutional biobank, enabling retrospective ELISA measurement of oxidative stress biomarkers. After applying the prespecified exclusion criteria (see below), the final analysis set comprised *n* = 90 patients. Wording such as “we measured” refers to retrospective assays on pre-existing biobank aliquots; no study-specific phlebotomy or prospective follow-up was undertaken.

Decompensated liver cirrhosis was defined by the presence of ascites, esophageal varices, or hepatic encephalopathy according to institutional protocols.

Exclusion criteria and rationale: We prespecified exclusions to reduce major sources of systemic oxidative stress confounding and HE misclassification: pregnancy; type 2 diabetes and/or metabolic syndrome; active autoimmune/inflammatory disease or ongoing systemic infection/sepsis; recent systemic corticosteroids or NSAIDs; and documented substance abuse. In addition, and specific to the biobank workflow, we excluded records with missing/invalid biobank consent or absence of a stored serum aliquot suitable for assays.

Screening log: Because this was a retrospective biobank study, a prospective screening log with per-criterion counts was not maintained. After internal audit, exact counts by exclusion category could not be reconstructed with sufficient fidelity. To avoid inaccurate reporting, we list all exclusion domains and report the final analyzed cohort (*n* = 90); table denominators (*n*/*N*) are provided where applicable.

Etiology and medication exposures: Cirrhosis etiology was abstracted from the discharge diagnosis and chart review and categorized as alcoholic, viral (HBV/HCV), MASLD, mixed, or other; when multiple etiologies were recorded, the primary category followed the attending physician’s attribution in the discharge summary. Medication exposures at admission were abstracted from medication orders/administration records and coded as present/absent within the first 48 h for: lactulose, rifaximin, spironolactone/other diuretics, albumin infusions, statins, and antioxidant supplements. Outpatient statin use was captured if documented as active on admission. Because documentation was partially missing in some charts, these covariates were analyzed descriptively.

### 2.2. Data Collection

#### 2.2.1. Sample Collection

Venous blood was drawn under routine clinical care at admission (no study-specific phlebotomy), then processed and stored at −80 °C in the institutional biobank; oxidative stress biomarkers were measured retrospectively from single-thaw aliquots. A standard procedure was followed to separate the clot by centrifugation (Hermle AG, Gosheim, Ba-den-Württemberg, Germany) at 3000× *g* for 10 min, no later than 4 h after collection.

Each patient’s serum sample cryotubes were labeled, sealed to prevent contamination, and kept at a temperature below −80 °C to allow for extended sample processing. Prior to assay, frozen samples were slowly thawed and centrifuged to remove precipitates. Also, a frozen sample was used for each ELISA kit from Elabscience (Houston, TX, USA).

#### 2.2.2. Clinical Chemistry and Hematology Platforms

Routine serum chemistry (AST/ALT, ALP, GGT, bilirubin, albumin, urea, creatinine, glucose, electrolytes, proteins, LDH, lipase, amylase, CRP) was performed on an automated clinical chemistry analyzer (e.g., Cobas c-series, Roche, Basel, Switzerland). Immunoassays, when applicable, were run on Cobas e411. Complete blood count was obtained on Abbott Alinity (5-part differential). Admission ammonia (NH_3_) levels were not systematically measured in routine practice and were therefore unavailable for robust analysis. A complete blood count (CBC) was performed using peripheral venous blood collected in vacutainer tubes containing ethylene-diamine-tetra-acetic acid (EDTA) as an anticoagulant. Utilizing flow cytometry and Coulter’s principle, we obtained an extended leukocyte formula of 5 diff (Alinity Abbott, Abbott Park, IL, USA) and determined the hemoleucogram markers: hemoglobin (Hb), Ht, white blood cells/leukocytes (WBCs), neutrophils (NEUs), lymphocytes (LYMs), monocytes (MONs), platelets (PLTs), the erythrocyte distribution width (RDW), and the mean corpuscular volume (MCV).

ESR was conducted by the Westergren method (ESR tubes, Becton Dickinson, Franklin Lakes, NJ, USA).

#### 2.2.3. Immunological Assessment

We used the Enzyme-Linked Immunosorbent Assay (ELISA) method at the University of Medicine and Pharmacy of Craiova’s Immunology Laboratory to measure serum oxidative stress mediator levels.

Commercial tests for each mediator were provided by the manufacturer, Elabscience (Houston, TX, USA): 8-epi-PGF2α (Cat.: E-EL-0041; sensitivity: 9.38 pg/mL; detection range: 15.63–1000 pg/mL), MDA (Cat.: E-EL-0060; sensitivity: 18.75 ng/mL; detection range: 31.25–2000 ng/mL).

The manufacturer’s instructions and recommended procedures were followed during the dilutions and processing steps. The procedures utilized a standard optical analyzer (Asys Expert Plus UV G020 150 Microplate Reader, ASYS Hitech GmbH, Eugendorf, Austria) with a wavelength of 450 nm.

#### 2.2.4. Pre-Analytical Handling

Venous blood was drawn under routine clinical conditions in the morning. Serum was separated within ≤4 h of collection, visually inspected, aliquoted (0.5–1.0 mL), and stored at −80 °C in the institutional biobank. For ELISA, aliquots were thawed once and cleared by brief centrifugation; samples with visible hemolysis, lipemia, or icterus were avoided. All oxidative stress assays were performed from single freeze–thaw aliquots.

#### 2.2.5. Assays & Units

Malondialdehyde (MDA) was quantified by ELISA and is reported in μmol/L equivalents derived from kit units (ng/mL) using the molecular weight of malondialdehyde (72.06 g/mol). Raw ng/mL values are available on request. For comparability with prior literature, we also provide µmol/L equivalents using the molecular weight of malondialdehyde (72.06 g/mol): µmol/L = (ng/mL) ÷ 72.06 (since 1 ng/mL = 1 µg/L and 1 µmol/L = 72.06 µg/L). Example: 180 ng/mL ≈ 2.50 µmol/L.

#### 2.2.6. Etiology and Medication Exposure

Etiology of cirrhosis was abstracted from the electronic medical record and hepatology notes and classified as alcoholic, viral (HBV and/or HCV), MASLD (metabolic dysfunction–associated steatotic liver disease), mixed (≥2 etiologies), or other/cryptogenic. When multiple etiologies were documented, cases were labeled mixed; when etiology could not be ascertained reliably, cases were labeled cryptogenic.

Medication exposure was captured at admission and within 48 h (or up to the time of serum sampling, if earlier) for agents known to influence oxidative stress or encephalopathy course: lactulose, rifaximin, diuretics (spironolactone and/or furosemide), intravenous albumin, statins, and antioxidants. Exposures were recorded as present/absent; denominators reflect available data where occasional missingness occurred.

Terminology and classes. Throughout the manuscript, Child–Pugh class refers to the conventional categories B and C derived from the original Child–Pugh score.

Severity definitions and operationalization: Hepatic encephalopathy (HE) was categorized as mild (West Haven 0–I; includes absent), moderate (West Haven II), and severe (West Haven III–IV). Ascites was graded as: grade 1 = mild (often detectable only by ultrasound), grade 2 = moderate, and grade 3 = severe/tense. Throughout the manuscript, both HE and ascites are treated as three-level ordinal variables and reported as mild, moderate, and severe. Where a binary sensitivity analysis is presented for ascites, it is explicitly labeled severe/tense (grade 3) vs. non-tense (grades 1–2) or present vs. absent, as appropriate.

Severity scores: In addition to Child–Pugh class, we computed the MELD-Na score using the OPTN/UNOS algorithm with standard bounds (bilirubin, INR, and creatinine values < 1.0 set to 1.0; creatinine capped at 4.0 mg/dL; serum sodium bounded to 125–137 mmol/L; natural logarithms used). Dialysis status within 7 days was not systematically captured in this retrospective extraction; therefore, the dialysis override was not applied. We did not calculate MELD 3.0 because sex was not available in the analytic spreadsheet; this is acknowledged as a study limitation.

### 2.3. Statistical Analysis

Statistical analyses were performed using IBM SPSS Statistics v. 26.0 (IBM Corp., Armonk, NY, USA). For descriptive analysis, continuous variables were reported both as mean ± standard deviation and median with interquartile range, depending on distribution. Categorical variables were expressed as counts and percentages. Sample size: No a priori sample-size calculation was performed; the cohort size (*n* = 90) was determined by the availability of eligible biobank cases in the study window. Analyses are framed as exploratory.

Sample size and power: No a priori power calculation was performed due to the retrospective design. As a reference, detecting small-to-moderate effects in χ^2^ comparisons typically requires substantially larger samples than available here; therefore, the present analyses are hypothesis-generating and precision is limited.

The normality of continuous variables was tested with the Shapiro–Wilk test and visual inspection. Group comparisons employed the Mann–Whitney U test for non-normal continuous data and the Chi-square test for categorical data. Associations between oxidative stress biomarkers (MDA, 8-iso-PGF2α) and both clinical severity scores (Child–Pugh, encephalopathy, ascites) and laboratory parameters (albumin, INR, bilirubin, creatinine) were examined using Spearman’s rank correlation coefficient.

A simplified composite score was developed to evaluate the joint predictive value of age and oxidative stress on cirrhosis severity. Patients older than 60 years and with MDA ≥ group median (µmol/L) were assigned two points; those meeting one or none of these criteria received 1 or 0 point(s). The association between this composite score and clinical severity (Child–Pugh B vs. C; severe encephalopathy [grade 3] vs. grades 1–2) was evaluated using the Chi-square test. This composite score was conceived as an exploratory, hypothesis-generating tool and not as a validated clinical model. Statistical significance was set at two-tailed *p* < 0.05, with 95% confidence intervals reported for key estimates.

### 2.4. Ethical Considerations

According to the European Union Guidelines (Declaration of Helsinki), the study received the approval of the Institutional Ethics Committee of the University of Medicine and Pharmacy of Craiova (registration no. 225/27 August 2024). In our institution, patients provide written broad consent at admission for biobanking and future research use of de-identified samples and clinical data; only cases with valid consent were eligible. No additional procedures were performed for the present study.

## 3. Results

### 3.1. Descriptive Analysis of the Patient Group

#### 3.1.1. Gender Distribution and Rationale for Age Stratification

A total of 90 patients diagnosed with liver cirrhosis were included in the analysis. The sex distribution revealed a predominance of male patients (*n* = 53, 58.9%) compared to females (*n* = 37, 41.1%). The age of the patients ranged from 36 to 76 years, with a median age of 59.5 years (IQR: 51–66), with a range of 36–76 years. However, the Shapiro–Wilk test showed a significant deviation from normality (W = 0.958, *p* = 0.005), suggesting a skewed distribution. This non-normality justified the use of non-parametric statistical methods for subsequent analyses.

- To account for the potential impact of age on disease severity, patients were stratified into four clinically and pathophysiologically relevant age classes. This classification was performed for exploratory purposes, reflects local clinical practice, and is not based on internationally validated cut-offs; it should therefore be interpreted as descriptive. Importantly, age stratification was not combined with liver function status in inferential analyses. Class 1 (<50 years): younger patients with higher physiological liver reserve and lower incidence of metabolic comorbidities.
- Class 2 (50–59 years): transitional group, typically exposed to emerging risk factors for fibrosis progression.
- Class 3 (60–69 years): older adults with increased risk of portal and renal complications.
- Class 4 (≥70 years): elderly patients with reduced hepatic reserve and multiple comorbidities, often requiring individualized therapeutic decisions.

Table 1 summarizes the demographic and clinical distribution across age classes. A male predominance was noted in classes 2 and 3, while gender proportions were more balanced in classes 1 and 4.

Across the four age classes (<50, 50–59, 60–69, ≥70), the distributions of gender, Child–Pugh class, hepatic encephalopathy severity (West Haven mapping), and ascites grade (IAC mapping) did not differ significantly (Chi-square *p* = 0.099, 0.795, 0.895, and 0.838, respectively). MELD-Na distribution. MELD-Na was available for all 90 participants, with a median of 20.9 (IQR 11.7–24.1). As expected, MELD-Na was higher in Child–Pugh class C than B (22.6 [21.8–26.5] vs. 11.0 [9.5–17.4]; Mann–Whitney U = 184; *p* < 0.001).

From a clinical perspective, liver function impairment—as assessed by the Child–Pugh classification—was more advanced in older patients. In classes 3 and 4, a higher proportion of individuals were categorized as Child–Pugh class C, consistent with advanced hepatic decompensation. This finding is aligned with prior evidence suggesting age-related deterioration in hepatic functional reserve.

The distribution of hepatic encephalopathy and ascites further illustrates the progression of liver disease with age. Severe ascites was present in 40/90 (44.4%), moderate ascites in 26/90 (28.9%), and mild ascites in 24/90 (26.7%). Hepatic encephalopathy grades were distributed as follows: grade 1 in 31/90 (34.4%), grade 2 in 28/90 (31.1%), and grade 3 in 31/90 (34.4%). These patterns support the hypothesis that age is associated with more severe clinical manifestations, although the presence of advanced features in younger groups indicates that age alone is not a sufficient determinant of severity.

The clinical and demographic distribution by age class reflects a heterogeneous picture, with significant differences supporting the usefulness of this stratification. In all four groups, the proportion of male patients was higher than the proportion of female patients, a trend slightly accentuated in the intermediate classes (50–69 years). This profile suggests a possible increased vulnerability of the male population to the aetiology and progression of liver cirrhosis, which merits further investigation.

From a functional point of view, the severity of liver impairment, as expressed by the Child–Pugh score, seems to correlate with increasing age. Thus, patients in classes 3 and 4 more frequently presented with stage C, indicating advanced decompensation of liver function and a low reserve of adaptation to physiological stress. This progressive course is consistent with the literature, which describes an increased risk of severe complications with advancing age.

As regards clinical manifestations of decompensation, moderate or severe hepatic encephalopathy was more common in patients aged 60 years or older, while absent or minimal forms were found mainly in younger classes. Severe ascites was also predominantly associated with advanced age classes, suggesting that massive peritoneal fluid accumulation may represent an indirect marker of deterioration of liver function in the context of advanced age. However, statistical testing revealed no significant differences across age groups regarding gender distribution (*p* = 0.099), Child–Pugh classification (*p* = 0.795), hepatic encephalopathy (*p* = 0.895), or ascites (*p* = 0.838). These findings suggest that, in this cohort, cirrhosis severity and decompensatory clinical manifestations were not significantly determined by age.

#### 3.1.2. Age Distribution of Biochemical Markers

To further explore the relationship between age and the degree of hepatic or extrahepatic dysfunction, we analyzed the distribution of key biochemical parameters across the four age classes. These included liver-related markers (total and direct bilirubin, albumin, INR, transaminases), renal function indicators (urea, creatinine), and other metabolic, hematologic, and enzymatic variables. A progressive increase in total and direct bilirubin levels was observed in the older age classes, particularly in class 4 (≥70 years), accompanied by a slight but consistent decline in serum albumin. This trend is indicative of deteriorating hepatocellular function and reduced hepatic reserve with advancing age. INR values also showed a tendency to rise in classes 3 and 4, suggesting impaired hepatic synthesis of coagulation factors. In contrast, transaminase levels (ALT and AST) remained relatively stable across groups, though ALT showed a slight increase in the oldest group, which may reflect ongoing hepatocellular injury or inflammation.

Renal parameters (urea and creatinine) did not differ significantly between classes, although creatinine values tended to be higher in older patients, possibly reflecting subclinical age-related renal impairment. Electrolytes (sodium, potassium), total protein, and markers of enzymatic cytolysis (LDH, lipase, amylase) showed minimal variation across age groups and no clear trends.

The hematologic profile revealed a modest age-related decline in hemoglobin and hematocrit levels, which is consistent with anemia of chronic disease frequently observed in advanced cirrhosis. Platelet counts were uniformly low across all groups, reflecting portal hypertension and hypersplenism, with no clear association with age.

Taken together, these findings suggest that advancing age is associated with a progressive impairment in liver function, as evidenced by elevated bilirubin and INR and decreased albumin levels. While not all differences reached statistical significance, the cumulative pattern is pathophysiologically consistent with age-related hepatic decompensation and supports the clinical stratification applied in this study.

Statistical analysis using the Kruskal–Wallis test revealed no significant differences between age groups for any of the biochemical parameters assessed (all *p* > 0.05). For example, hematocrit (*p* = 0.124), hemoglobin (*p* = 0.402), total bilirubin (*p* = 0.503), INR (*p* = 0.509), and creatinine (*p* = 0.812) showed no significant variation across the four strata. Although descriptive trends suggested higher bilirubin and INR values and lower albumin in elderly patients, these differences did not reach statistical thresholds. The absence of significance can be attributed to several factors, including the relatively small sample size within each age stratum, the heterogeneous etiology of cirrhosis, and the advanced disease profile observed across the entire cohort. These results are consistent with international evidence indicating that, in decompensated cirrhosis, many biochemical markers plateau regardless of age, reflecting global hepatocellular dysfunction rather than age-specific progression.

Information on cirrhosis etiology and concomitant therapies was partially available. Among the documented cases, alcoholic cirrhosis predominated, followed by viral-related forms (HBV and HCV), with fewer metabolic or other causes. Data on alcohol consumption and on concomitant treatments such as albumin or statins were inconsistently recorded and therefore only partially analyzed.

Table 2 presents the median values and interquartile ranges (IQRs) for each parameter by age group.

#### 3.1.3. Distribution of Clinical Scores by Age Class

The clinical severity of liver disease was assessed in relation to age by analyzing three key parameters: the Child–Pugh classification, the grade of hepatic encephalopathy (HE), and the severity of ascites. Given the ordinal nature of these clinical scores and the non-Gaussian distribution of variables, associations were examined using non-parametric tests, and results are summarized in Table 1.

Across all age groups, Child–Pugh classes B and C were predominant, with a progressive increase in class C in older patients. In the ≥70 years group (class 4), two-thirds of patients (66.7%) were classified as Child–Pugh class C, indicating a markedly reduced hepatic reserve and more advanced decompensation in elderly individuals. This observation is consistent with the natural history of cirrhosis, where disease progression and comorbidities tend to worsen with age.

Across age classes, the distribution of HE severity did not differ significantly (χ^2^, *p* = 0.895; Table 1). Numerically, severe HE (West Haven III–IV) occurred more often in Classes 1–2 than in Class 3; however, these differences were not statistically significant.

Taken together, these distributions indicate that advanced decompensation features are present across age classes; however, no statistically significant differences by age class were detected for Child–Pugh, HE, or ascites.

Overall, these clinical patterns confirm that advanced forms of liver cirrhosis were present across the entire cohort, with greater frequency and severity observed in older patients. Nonetheless, the occurrence of decompensatory features among younger individuals highlights the multifactorial nature of cirrhosis progression and reinforces the need for individualized clinical evaluation beyond age stratification alone.

The distribution of clinical scores supports the idea that decompensation of cirrhosis can occur in both elderly and younger patients, with clinical variability emphasising the need for integrated assessment of disease severity.

Analysis of the clinical scores of the entire group of patients with cirrhosis of the liver shows a severe disease profile, characterised by a predominance of advanced grades in the Child–Pugh score. No patient was classified in class A, and the majority of patients were classified in class C (57.8%), reflecting a severely compromised liver reserve at the time of evaluation. Almost uniformly distributed, hepatic encephalopathy was present in all three stages analysed, with a similar frequency for grades 1 and 3 (34.4%), suggesting variable but frequent neurological impairment among patients. As regards ascites, the severe (“large”) form was the most frequent (44.4%), indicating marked clinical decompensation in more than one third of cases. These parameters support the conclusion of an advanced cirrhosis, with systematised clinical manifestations relevant for staging the disease in current practice. For a synthetic understanding of the clinical severity in the whole studied group, we have centralised the distribution of relevant scores in Table 3.

Statistical testing of the association between patient age and Child–Pugh score showed no significant differences between the analysed classes. Although the descriptive analysis suggested a trend of worsening liver severity in the older age groups (with a higher proportion of patients in class C at ≥70 years), the Chi-square test applied to the distribution of Child–Pugh scores according to age classes did not demonstrate a significant association (Chi^2^ = 1.03, df = 3, *p* = 0.795). These results indicate that the clinical severity of cirrhosis, as assessed by the Child–Pugh score, did not vary significantly by age in the analysed group, suggesting that younger patients may also have advanced forms of the disease.

### 3.2. Association of Oxidative Stress Markers with Clinical Parameters and Liver Scores

#### 3.2.1. Distribution of Child–Pugh Score According to Quartiles of Oxidative Stress Markers

Considering the potential role of oxidative stress in the progression of liver disease and impairment of hepatocellular function, we examined the distribution of Child–Pugh classes B and C according to the quartiles of the two oxidative stress biomarkers included in the study: malondialdehyde (MDA) and 8-epi-prostaglandin F2α (8-iso-PGF2α). Quartiles were generated based on the distribution of measured values for each marker, allowing for a comparative analysis of liver dysfunction severity across subgroups with varying oxidative stress levels.

As shown in Table 4, median MDA levels were 2.45 [1.98–3.05] μmol/L in Child–Pugh B and 2.67 [2.10–3.20] μmol/L in Child–Pugh C, with no significant difference (Mann–Whitney U = 626.0, *p* = 0.331). For 8-iso-PGF2α, median values were 250.1 [210.0–295.0] pg/mL in Child–Pugh B and 255.8 [220.0–310.0] pg/mL in Child–Pugh C (*p* = 0.784). While oxidative stress markers did not significantly differentiate between Child–Pugh stages, albumin was significantly lower and INR significantly higher in class C, consistent with advanced hepatic decompensation. Overall, while oxidative stress biomarkers do not exhibit a direct or significant association with Child–Pugh classification in this cohort, a potential relationship cannot be ruled out. Further investigation among a larger population or with the integration of additional markers may better clarify this association.

#### 3.2.2. Hepatic Encephalopathy According to Oxidative Stress Markers

Hepatic encephalopathy (HE) is a common neuropsychiatric complication of liver cirrhosis, associated with the accumulation of toxic metabolites, mainly ammonia, in the context of impaired hepatic clearance function and portosystemic shunting. The severity of manifestations ranges from subtle cognitive impairment to hepatic coma and is clinically classified into several grades. In our database, the encephalopathy score was coded from 1 to 3, reflecting the degree of neurological impairment: 1—mild/subclinical forms; 2—moderate forms; and 3—severe or clinically manifest forms. These were mapped to West Haven grades 0–IV (Absent/mild = 0–I; Moderate = II; Severe = III–IV; no grade IV cases were observed).

As the patient’s neuropsychiatric status is susceptible to changes in oxidative homeostasis and alterations in cerebral microcirculation, we analysed the distribution of hepatic encephalopathy score according to quartiles of MDA and 8-iso-PGF2α values. The aim was to assess whether patients with higher oxidative stress have more severe forms of encephalopathy.

As shown in Table 5, median MDA values differed significantly across encephalopathy grades (Kruskal–Wallis H = 7.70, *p* = 0.021), indicating that higher oxidative stress was associated with more severe neuropsychiatric impairment. By contrast, 8-iso-PGF2α levels showed no significant variation across grades (H = 1.86, *p* = 0.395). These results suggest that MDA, but not 8-iso-PGF2α, may be linked to the severity of hepatic encephalopathy in cirrhotic patients. This analysis suggests that, in the context of small cohorts or unevenly prevalent HD manifestations, oxidative stress markers may reflect individual variation but are not, as yet, independent predictors of hepatic encephalopathy severity. Such a relationship is likely to become evident only in a prospective, controlled design with standardised neurocognitive assessment correlated with central and peripheral biomarkers.

#### 3.2.3. Ascites According to Oxidative Stress Markers

Primary analyses used the International Ascites Club three-level definition (Low/Moderate/High = grades 1/2/3); we additionally report a clearly labeled binary sensitivity analysis (present vs. absent). Analyses of ascites distribution according to quartiles of MDA and 8-iso-PGF2α values followed possible relationships between the intensity of oxidative stress and fluid retention status.

As shown in Table 6, no significant differences in oxidative stress markers were observed across ascites severity categories (low, moderate, high). Median MDA values did not vary significantly (Kruskal–Wallis H = 1.58, *p* = 0.453), and 8-iso-PGF2α showed a similar non-significant pattern (H = 0.48, *p* = 0.828). Binary comparisons (ascites present vs. absent) confirmed the absence of statistical association. These results suggest that oxidative stress markers do not independently reflect fluid accumulation status in cirrhotic patients. These results suggest that, in the cohort analysed, oxidative stress is not an isolated determinant of ascites. Its influence is likely to be modulated by multiple factors, such as renal function, systemic inflammatory profile, and vasodilatory alterations characteristic of advanced cirrhosis. In the context of a multifactorial approach, oxidative markers could be integrated into more complex predictive models, alongside standardised clinical and biological parameters.

#### 3.2.4. Child–Pugh Score About Oxidative Stress Markers

The Child–Pugh score remains a cornerstone tool for assessing cirrhosis severity, integrating both clinical (ascites, encephalopathy) and biochemical parameters (bilirubin, albumin, INR). In this study, we analysed whether oxidative stress markers differ between Child–Pugh classes B and C when considered as continuous variables.

As shown in Table 7, median MDA values were 2.45 [1.98–3.05] μmol/L in class B and 2.67 [2.10–3.20] μmol/L in class C, with no significant difference (Mann–Whitney U = 626.0, *p* = 0.331). For 8-iso-PGF2α, levels were 250.1 [210.0–295.0] pg/mL in class B and 255.8 [220.0–310.0] pg/mL in class C (*p* = 0.784). In contrast, classical liver function markers showed highly significant differences: albumin was markedly reduced in class C (2.50 [2.40–2.70] g/dL vs. 3.20 [2.80–3.40] g/dL, *p* < 0.001), while INR was significantly increased (1.70 [1.50–1.80] vs. 1.30 [1.20–1.40], *p* < 0.001).

These results highlight that oxidative stress markers, unlike conventional biochemical indicators, do not discriminate between Child–Pugh stages. This suggests that systemic oxidative stress may plateau in advanced cirrhosis and reflect broader pathophysiological processes—including inflammation, fibrosis, and microvascular dysfunction—rather than directly mirroring the degree of hepatic functional reserve. Thus, while oxidative stress is central to cirrhosis progression, its serum markers (MDA, 8-iso-PGF2α) may have limited discriminative power once patients reach decompensated stages.

#### 3.2.5. Continuous Correlations Between Marker Values and the Numerical Child–Pugh Score

To further investigate the relationship between oxidative stress and cirrhosis severity, we performed correlation analyses between continuous biomarker values and clinical parameters using Spearman’s rank test. As shown in Table 8, MDA values did not correlate significantly with the Child–Pugh score (ρ = −0.08, *p* = 0.47), encephalopathy grade (ρ = −0.01, *p* = 0.94), or ascites severity (ρ = −0.06, *p* = 0.59). Similarly, 8-iso-PGF2α levels were not associated with Child–Pugh score (ρ = 0.00, *p* = 0.98), encephalopathy (ρ = 0.00, *p* = 0.96) or ascites (ρ = −0.02, *p* = 0.84). The only near-significant association was a negative trend between 8-iso-PGF2α and total bilirubin (ρ = −0.18, *p* = 0.09).

These results indicate that neither MDA nor 8-iso-PGF2α strongly correlates with global indices of cirrhosis severity. This pattern supports the concept that serum oxidative stress biomarkers, although elevated in advanced disease, may reflect systemic variability and plateau effects rather than progressive deterioration captured by composite scores such as Child–Pugh. Thus, oxidative stress appears to contribute to cirrhosis pathophysiology in an indirect manner, alongside inflammation, fibrosis, and vascular dysfunction, rather than showing a simple linear relationship with disease stage.

#### 3.2.6. Integrative Interpretation of the Relationship Between Oxidative Stress and Liver Status

The overall analysis confirmed a consistently elevated oxidative stress profile in the studied cohort, as reflected by MDA and 8-iso-PGF2α values. However, the relationship between these biomarkers and conventional indices of liver severity was heterogeneous. No significant differences in biomarker levels were observed between Child–Pugh classes B and C, nor across categories of ascites, and correlation analyses with albumin, INR, bilirubin, creatinine, and age were also non-significant.

Importantly, MDA showed a significant association with hepatic encephalopathy grade (*p* = 0.021, Kruskal–Wallis), suggesting a potential link between systemic oxidative stress and the severity of neuropsychiatric impairment. In contrast, 8-iso-PGF2α did not demonstrate significant variation across encephalopathy grades.

Taken together, these results suggest that serum oxidative stress markers do not directly mirror the degree of hepatic functional reserve, but may still reflect clinically relevant complications such as encephalopathy. This supports the hypothesis that oxidative stress is an important pathogenic driver, yet its clinical expression is modulated by multiple interacting processes, including systemic inflammation, fibrogenesis, and vascular dysfunction. The plateau of marker values across Child–Pugh classes observed here may indicate that, in advanced cirrhosis, oxidative stress is already maximally expressed and loses discriminative power for staging, while remaining relevant for extrahepatic manifestations.

#### 3.2.7. Comparison of Clinico-Biological Parameters According to Child–Pugh Score Severity

To assess the differences between grades of liver severity, patients were subdivided according to the Child–Pugh score, limited in this group to grades B (code 2) and C (code 3). The Mann–Whitney U test was used to compare age, relevant biochemical parameters (ALTALT, AST, bilirubin, albumin, INR), and oxidative stress markers (MDA and 8-iso-PGF2α).

The median age of the patients in class C was slightly older compared to those in class B (64 vs. 59 years), but the difference did not reach statistical significance (*p* = 0.085). Classical liver parameters showed important variations: patients with Child–Pugh C score had significantly lower albumin levels (median 2.5 g/dL vs. 3.2 g/dL; *p* < 0.0001) and increased INR values (1.70 vs. 1.30; *p* < 0.0001), reflecting decompensation of hepatic synthesis. Total bilirubin also tended to be higher in class C (2.6 vs. 2.0 mg/dL), but without statistical significance (*p* = 0.117). Transaminases (AST, ALT) did not differ significantly between the two classes, suggesting that the degree of acute hepatocytic necrosis is not a reliable discriminator between stages of chronic severity.

As for oxidative stress markers, MDA and 8-iso-PGF2α values did not significantly differ between Child–Pugh B and C classes (*p* = 0.331 and *p* = 0.784), indicating the absence of a direct correlation between worsening liver function and the intensity of systemic oxidative stress in this group (Table 8).

#### 3.2.8. Correlations Between Oxidative Stress and the Severity of Liver Disease

Given the putative role of oxidative stress in the progression of liver damage, we aimed to assess the correlations between the levels of MDA and 8-iso-PGF2α markers and clinical and biological parameters relevant to disease severity. This analysis aimed to identify possible direct relationships between oxidative stress and the degree of liver decompensation in the context of cirrhosis.

Spearman’s non-parametric correlation analysis aimed to explore the relationship between oxidative stress markers (MDA and 8-iso-PGF2α) and the severity of cirrhosis, assessed by relevant clinico-biological parameters. No significant correlations were found between MDA values and Child–Pugh score (r = −0.06, *p* = 0.56), degree of hepatic encephalopathy (r = −0.10, *p* = 0.36), or severity of ascites (r = −0.13, *p* = 0.23). Also, correlations between MDA and classical biochemical parameters (albumin, INR, total bilirubin, and creatinine) were statistically insignificant, indicating a potential dissociation between oxidative stress levels and these variables in the analysed group. No significant correlation with age was observed for either biomarker (MDA: Spearman ρ = −0.09, *p* = 0.42; 8-iso-PGF2α: ρ = −0.03, *p* = 0.81; *n* = 90).

Similarly, 8-iso-PGF2α values showed no significant correlations with any of the clinical or paraclinical markers included in the analysis. The closest trend of association was observed between 8-iso-PGF2α and total bilirubin (r = −0.18, *p* = 0.09), but without reaching statistical significance, as seen in Table 9.

These results suggest that, in this group of patients with liver cirrhosis, oxidative stress markers (MDA and 8-iso-PGF2α) do not directly reflect clinical severity or degree of hepatic decompensation, which may be explained either by aetiological heterogeneity of the disease or by variable compensatory mechanisms involved in oxidative homeostasis.

#### 3.2.9. Exploration of a Composite Score Based on Age and Oxidative Stress Concerning Cirrhosis Severity

Starting from the biologically plausible assumption that advancing age, correlated with the accumulation of oxidative stress, may amplify the risk of liver decompensation, we aimed to construct a simplified composite score reflecting this interaction. The idea was to test, even in an exploratory manner, whether the association between advanced age and elevated levels of MDA (a marker of lipid oxidative stress) can differentiate patients with more severe forms of the disease. The cut-off of 60 years was selected in agreement with the clinical literature, which emphasises a marked deterioration of liver reserves with advancing age. Regarding MDA, the value was considered increased when it exceeded the group median, reflecting an intensification of oxidative stress in the upper half of the distribution.

##### Score Construction

The score was generated by assigning one point for each of the following conditions:

- Patient age > 60 years.
- MDA value ≥ group median (µmol/L).

Thus, patients who cumulated both characteristics (score = 2) were considered to have a biological and demographic profile associated with increased risk. For ease of statistical analysis, the score was subsequently recoded in a binary manner: score 2 = ‘positive’ (concomitant presence of both factors), score 0 or 1 = ‘negative’.

##### Association with Disease Severity

The composite score distribution was compared with two dimensions of liver severity:

- Child–Pugh classification (B vs. C), using the Chi-square test.
- Severity of hepatic encephalopathy (stage 3 vs. stages 1–2), using the same method.

Results showed a significant association between the composite score and Child–Pugh class C (*p* = 0.028), suggesting that patients with advanced age and increased MDA tend to manifest a more severe clinical form. In the case of severe hepatic encephalopathy, a trend of association was shown (*p* = 0.061), without reaching the statistical significance threshold. Group sizes and outcome rates are shown in Table 10.

Although the results should be interpreted with caution, given the exploratory nature of the score and the relatively modest sample size, they provide an interesting signal about the relevance of integrating simple but complementary biological factors. The observed association between the composite score and clinical severity indicates a possible synergistic role of age and oxidative stress in liver disease progression, opening potential directions for future, more elaborate predictive models. Such prospectively validated tools could contribute to early risk stratification in populations with advanced cirrhosis.

## 4. Discussion

The results obtained in this study highlight a severe clinical profile of liver cirrhosis among the analysed patients, characterised by a predominance of Child–Pugh B and C grades, with an increased frequency of complications such as hepatic encephalopathy and severe ascites. The age distribution of patients revealed relevant clinico-biological differences, particularly in the advanced age groups, where higher INR and bilirubin levels were observed, in parallel with a decrease in serum albumin, all indicating progressive liver damage. These findings are consistent with recent literature, which emphasises worsening of liver function with advancing age and accumulation of comorbidities [24].

Although the values of oxidative stress markers (MDA and 8-iso-PGF2α) were elevated in most cases, direct correlations with liver severity parameters did not reach statistical significance. This finding is consistent with international evidence, where these biomarkers have shown heterogeneous and often inconsistent associations with clinical severity in decompensated cirrhosis. Reporting such negative results remains valuable, as it refines current understanding and delineates the limited prognostic utility of these markers. Previous studies have suggested possible links between MDA and parameters of liver or neurocognitive dysfunction [25], but our results indicate that such associations could not be consistently demonstrated in this cohort. Importantly, our data showed that MDA levels increased significantly with encephalopathy severity (*p* = 0.021), suggesting that systemic oxidative stress may contribute to neuropsychiatric impairment in cirrhotic patients.

The markers utilised in our analysis—malondialdehyde (MDA) and 8-iso-PGF2α—have been frequently proposed in the literature as biomarkers of lipid peroxidation and systemic inflammation associated with liver dysfunction. Zheng et al. reported an association between elevated MDA levels and reduced grey matter volume in nonalcoholic cirrhosis, suggesting a relationship between oxidative stress and neurocognitive impairment observed in hepatic encephalopathy [14]. Experimental and clinical studies in recent years confirm that oxidative stress contributes to mitochondrial dysfunction, hepatic stellate cell activation and fibrogenesis, and is involved in the transition from steatohepatitis to cirrhosis and subsequently to hepatocellular carcinoma [24,26]. In an analysis of patients with metabolic liver disease, Ma et al. identified MDA as a relevant marker for the severity of liver damage in NAFLD and NASH, with a significant association between its levels and histological scores of inflammatory activity [26]. Delli Bovi et al. have also proposed the integration of MDA into risk stratification algorithms, given its correlation with systemic proinflammatory status [27]. 8-iso-PGF2α, although less widely used in current practice, is recognised as a specific biomarker for lipid oxidative stress, being detected in increased concentrations in patients with advanced liver damage, but with high interindividual variability [28,29]. A recently published prospective study showed that elevated levels of 8-iso-PGF2α in urine may predict the risk of hepatocarcinoma among patients with chronic liver disease. However, the clinical applicability of this marker remains to be validated [30]. In our study, neither MDA nor 8-iso-PGF2α levels differed significantly between Child–Pugh classes or across categories of ascites severity. These negative findings emphasise that oxidative stress markers appear to have limited value for staging cirrhosis severity in decompensated patients.

An original contribution of this study is the formulation and testing of a simplified composite score based on the association between age > 60 years and elevated MDA values (≥2.41 μmol/L). This score was significantly associated with clinical severity, being more common in Child–Pugh class C patients. These results suggest that biological ageing, together with systemic oxidative stress, may synergistically contribute to hepatic decompensation. However, since the score was derived and tested within the same cohort, it must be regarded as exploratory. External validation in larger, prospective cohorts is required before any clinical applicability can be considered. The idea of combining these parameters is supported by well-documented pathophysiological premises [24,28]. The Age + MDA composite was conceived as a minimal, hypothesis-generating signal-finding tool. In our cohort, age and MDA were not correlated (Spearman ρ = −0.09; *p* = 0.42; *n* = 90), suggesting complementary rather than redundant information; nonetheless, effect sizes were small, no internal validation was performed, and the composite is not intended for clinical use.

The analysis performed in this study showed that patients with age > 60 years and elevated MDA values were significantly more likely to be classified in Child–Pugh score class C than those without both factors. This association suggests that the synergy between age and oxidative stress may contribute to accelerated liver decompensation, overriding the influence of each factor individually. Interestingly, neither MDA nor age alone had a robust predictive power, but the combined score showed statistical significance, which reinforces the validity of this integrative model. Although the proposed score has a simple construction, its advantage lies in its feasibility for clinical application without requiring advanced testing or complex calculations. Thus, this tool could be valuable in resource-limited clinical settings where rapid risk stratification is required. Previous studies have suggested the usefulness of composite scores based on biological and age parameters in other liver pathologies. However, most of them have focused on extended scores (e.g., MELD or complex biochemical modelling) [27,31]. At the same time, it should be noted that validation of the score in a small sample and a retrospective design limits general conclusions about its applicability. However, the results obtained support the utility of exploring this approach in future studies, preferably prospective, including correlations with long-term outcome, survival, and the need for liver transplantation.

Taken together, our findings provide clinically useful insights. Oxidative stress biomarkers did not add discriminatory power for prognostic stratification by Child–Pugh class or ascites, but MDA was associated with encephalopathy severity. These results highlight that oxidative stress markers should not be applied indiscriminately for staging, yet they may have a role in predicting or monitoring specific complications where conventional scores are less granular.

The present study should also be interpreted in the light of methodological limitations which, although inherent to a retrospective design, may influence the generalisability of the results. Firstly, the sample size analysed was relatively modest, which may reduce the statistical power of the tests applied, especially in the context of subgroup analyses or exploratory evaluations. This limitation is particularly relevant in the case of relationships between oxidative stress markers and clinical severity scores, where the observed differences were often suggestive but not always supported by statistical significance. It should also be noted that the patients included in the study presented with liver cirrhosis of heterogeneous aetiology, without a clear stratification according to cause (viral, alcoholic, metabolic, etc.). This lack of etiological homogeneity may be a source of variability in oxidative response and clinical severity, potentially influencing the relationships identified in the analysis. At the same time, the cross-sectional nature of the study does not allow us to assess the predictive value of MDA and 8-iso-PGF2α markers on long-term outcome, complications, or survival, but only to explore a possible correlation at the time of clinical evaluation. As for the proposed composite score, it was derived and tested in the same group of patients without an external validation step. Even if the results obtained are promising, statistical overcorrelation cannot be excluded, and the applicability of the score in other populations remains unproven. Also, the interpretation of oxidative stress marker values should be made with caution, considering the possible influences of other systemic clinical conditions or treatments administered, which could not be fully controlled in this retrospective design.

We report MELD-Na; MELD 3.0 was not computed because sex was not recorded in the analytic dataset, although albumin and other components were available. This omission is unlikely to change the main conclusions but is acknowledged as a limitation.

The results provide preliminary, hypothesis-generating support for a link between oxidative stress, age, and the severity of liver decompensation. The simplicity of the proposed score and the feasibility of its application in practice justify further research in this direction. Prospective studies in larger and homogeneous cohorts could clarify to what extent such simple but integrated scores can contribute to risk stratification and to predicting the clinical course of patients with liver cirrhosis.

The role of oxidative stress in liver diseases has been thoroughly examined, with research highlighting the influence of reactive oxygen species (ROS) and reactive nitrogen species (RNS) in the development of conditions such as ARLD, non-alcoholic fatty liver disease (NAFLD), viral hepatitis, and hepatocellular carcinoma (HCC) [5,9].

The liver serves as the primary location for alcohol metabolism and is among the initial sites affected by alcohol-induced injuries. In ARLD, the metabolic processing of alcohol necessitates the activation of the cytochrome P450 2E1 (CYP2E1) isoform, which is responsible for the generation of reactive oxygen species (ROS). Reactive oxygen species (ROS) can interact with fatty acids derived from lipids, leading to the formation of various peroxides. These compounds can interact with proteins and DNA, forming adducts that lead to structural and functional changes in liver cells, ultimately resulting in cell death signaling. The alternative liver processing of alcohol via the alcohol dehydrogenase reaction produces acetaldehyde, a reactive intermediate that can interact with proteins and DNA, leading to the formation of adducts that exacerbate hepatocellular damage [22].

Alcohol consumption modifies antioxidant systems responsible for the removal of reactive oxygen species via intricate signaling pathways. Ultimately, reactive oxygen species (ROS) can result in significant liver fibrosis and cirrhosis through the activation of hepatic stellate cells, which play a role in the accumulation of extracellular matrix in the liver [12].

The alteration of the pro-oxidant/antioxidant balance was demonstrated in liver and blood samples of patients through various techniques, including direct quantification of reactive oxygen species (ROS) and reactive nitrogen species (RNS), identification of tissue storage of oxidative and nitrosative stress markers, measurement of lipid, protein, and DNA oxidation products, as well as assessments of individual antioxidants and total antioxidant capacity.

Interpretation for discordant HE associations (MDA vs. 8-iso-PGF2α). A plausible explanation for the discrepant behavior is both analytic and biological. Serum 8-iso-PGF2α measured by immunoassay is more susceptible to pre-analytical/assay variability (matrix effects, cross-reactivity) and often performs better when assessed as urinary isoprostanes or by LC/GC-MS reference methods. In contrast, MDA reflects a broader aldehyde burden from lipid peroxidation and protein adducts, which may track neurocognitive vulnerability more closely in decompensated cirrhosis. Additionally, in advanced disease, lipid peroxidation may plateau across clinical strata (ceiling effect), while heterogeneity in inflammation, cholestasis, renal function, nutrition, and medications can differentially influence these markers. Together, these factors can yield a detectable signal for MDA but a weaker gradient for serum 8-iso-PGF2α with respect to HE.

This study has several limitations. First, the stratification into four age classes was exploratory and not based on validated clinical thresholds, so results should be interpreted with caution. Second, information on cirrhosis etiology and concomitant therapies, including alcohol consumption, albumin, and statins, was incompletely available due to the retrospective design, which may influence oxidative stress profiles. The sample size was determined by case availability in the biobank, without a priori power calculation; therefore, findings should be interpreted as hypothesis-generating. Residual confounding. Etiology and medication exposures were incompletely captured and not fully standardized in this retrospective extraction; therefore, residual confounding cannot be excluded, and adjusted models were not pursued to avoid unstable estimates. Finally, no follow-up data were available, which prevented the evaluation of short- and long-term outcomes. Multiple non-hepatic and treatment-related drivers of oxidative stress (e.g., systemic inflammation/endotoxemia, alcohol use or recent withdrawal, cholestasis-related mitochondrial dysfunction, iron overload, renal dysfunction, sarcopenia/micronutrient deficits, intercurrent infections or GI bleeding, and drug exposures such as diuretics or dehydration from lactulose) may persist despite clinical stratification, potentially diluting biomarker–phenotype contrasts in this retrospective cohort.

## 5. Conclusions

This study provides a detailed clinical and biochemical characterization of patients with decompensated cirrhosis in a large regional cohort, reflecting the real-world profile of advanced disease in our population. Elevated levels of oxidative stress markers (MDA and 8-iso-PGF2α) were observed, but no significant correlations with clinical severity were identified. These negative findings are consistent with international evidence and contribute valuable data by delineating the limited prognostic role of such markers in decompensated disease.

An exploratory composite score combining age > 60 years and elevated MDA values was significantly associated with advanced Child–Pugh class. While this observation is hypothesis-generating and requires validation in larger, prospective cohorts, it suggests that the interaction between biological ageing and systemic oxidative stress may be relevant in hepatic decompensation.

Taken together, our results emphasize both the complexity of cirrhosis and the importance of reporting negative as well as exploratory findings. By providing regional data aligned with international research, this study contributes to refining prognostic approaches and highlights directions for future investigation.

## Figures and Tables

**Table 1:** Demographic and clinical distribution by age classes (Class 1: <50; Class 2: 50–59; Class 3: 60–69; Class 4: ≥70). Hepatic encephalopathy mapped to West Haven; ascites mapped to IAC.

Parameter	Values	Age (Years)							
		<50		50–59		60–69		>70	
		*N*	%	*N*	%	*N*	%	*N*	%
Gender	Female	6	33.33%	9	33.33%	3	9.09%	3	25.00%
	Male	12	66.67%	18	66.67%	30	90.91%	9	75.00%
Child–Pugh	B	7	38.89%	11	40.74%	16	48.48%	4	33.33%
	C	11	61.11%	16	59.26%	17	51.52%	8	66.67%
Encephalopathy	Mild	5	27.78%	9	33.33%	13	39.40%	4	33.33%
	Moderate	7	38.89%	7	25.93%	11	33.33%	3	25.00%
	Severe	6	33.33%	11	40.74%	9	27.27%	5	41.67%
Ascites	Severe	8	44.44%	11	40.74%	15	45.46%	6	50.0%
	Mild	5	27.78%	6	22.22%	11	33.33%	2	16.67%
	Moderate	5	27.78%	10	37.04%	7	21.21%	4	33.33%

Notes. Age classes (Class 1: <50 years; Class 2: 50–59 years; Class 3: 60–69 years; Class 4: ≥70 years). Hepatic encephalopathy categories map to West Haven: Mild = Grade 0–I (includes absent); Moderate = Grade II; Severe = Grades III–IV. Ascites categories map to International Ascites Club (IAC): Mild = Grade 1; Moderate = Grade 2; Severe/tense = Grade 3. Test: Chi-square across the four age classes; *p*-values: Gender *p* = 0.099; Child–Pugh *p* = 0.795; Hepatic encephalopathy *p* = 0.895; Ascites *p* = 0.838. Data are shown as *n*/*N* (%); denominators may vary due to sporadic missingness.

**Table 2:** Baseline laboratory parameters by age classes (Class 1: <50; Class 2: 50–59; Class 3: 60–69; Class 4: ≥70 years).

Parameter	Age (Years)			
	<50	50–59	60–69	>70
Alanine aminotransferase (ALT, U/L)	38.50 (27.25–64.75)	44.00 (27.00–67.50)	44.00 (27.00–68.00)	62.50 (29.25–76.25)
Aspartate aminotransferase (AST, U/L)	79.50 (52.00–87.00)	78.00 (44.50–100.00)	72.00 (36.00–113.00)	85.50 (77.25–95.50)
Alkaline phosphatase (ALP, U/L)	106.00 (89.75–132.00)	107.00 (69.00–132.00)	113.00 (97.17–136.00)	114.00 (95.12–132.25)
Direct bilirubin (mg/dL)	2.52 (0.54–3.75)	2.27 (0.48–3.55)	1.83 (0.30–2.79)	2.80 (1.77–4.40)
Total bilirubin (mg/dL)	3.97 (1.34–4.80)	3.88 (1.08–5.75)	3.10 (0.90–5.71)	4.38 (3.33–6.06)
Albumin (g/dL)	2.55 (2.40–2.77)	2.50 (2.45–2.80)	2.70 (2.50–3.00)	2.65 (2.35–2.90)
International normalized ratio (INR)	1.44 (1.30–1.60)	1.47 (1.31–1.60)	1.40 (1.18–1.72)	1.56 (1.41–1.70)
Urea (mg/dL)	45.00 (36.25–65.75)	40.66 (29.48–66.50)	50.00 (40.00–74.00)	45.00 (40.00–58.50)
Creatinine (mg/dL)	1.15 (1.03–1.47)	1.04 (0.95–1.46)	1.20 (1.00–1.41)	1.20 (0.89–1.62)
Glucose (mg/dL)	108.00 (95.00–122.00)	101.00 (94.50–122.00)	100.00 (94.00–122.00)	119.00 (104.25–133.75)
Sodium (mmol/L)	136.00 (131.25–141.00)	136.00 (133.00–140.50)	135.00 (131.00–141.00)	131.50 (127.00–137.00)
Potassium (mmol/L)	4.25 (3.92–4.60)	4.10 (3.75–4.60)	4.00 (3.60–4.50)	3.90 (3.12–4.35)
Total protein (g/dL)	5.05 (4.50–5.28)	5.00 (4.50–5.90)	4.90 (4.50–5.50)	5.05 (4.72–5.50)
Lactate dehydrogenase (LDH, U/L)	121.00 (110.00–130.00)	122.00 (107.50–134.00)	125.00 (115.00–138.00)	122.00 (119.75–129.25)
Lipase (U/L)	52.00 (38.75–74.25)	52.00 (39.50–68.00)	52.00 (42.00–69.00)	64.50 (52.00–100.50)
Amylase (U/L)	66.50 (52.00–82.75)	63.00 (46.50–84.00)	73.00 (56.00–88.00)	68.50 (44.00–80.75)
Hemoglobin (HGB, g/dL)	9.43 (8.30–12.15)	10.40 (8.45–12.25)	10.70 (8.90–12.00)	9.10 (8.05–11.62)
Hematocrit (%)	0.36 (0.31–19.46)	0.36 (0.32–13.43)	0.32 (0.30–0.35)	0.33 (0.33–0.36)
White blood cells (WBC, ×10^9^/L)	3380.00 (85.10–6155.00)	85.40 (69.50–3905.00)	85.40 (63.50–5420.00)	2839.50 (73.90–8130.00)
Neutrophils (%)	0.53 (0.46–0.55)	0.52 (0.46–0.55)	0.52 (0.48–0.56)	0.55 (0.53–0.56)
Lymphocytes (%)	0.31 (0.26–0.36)	0.32 (0.26–0.36)	0.31 (0.25–0.36)	0.32 (0.28–0.35)
Monocytes (%)	0.11 (0.10–0.15)	0.11 (0.10–0.13)	0.12 (0.11–0.14)	0.11 (0.11–0.15)
Platelets (PLT, ×10^9^/L)	57,500.00 (1532.50–111,750.00)	2070.00 (1115.00–59,900.00)	56,000.00 (1720.00–123,000.00)	60,000.00 (43,400.00–67,250.00)
Red cell distribution width (RDW, %)	0.17 (0.14–0.18)	0.16 (0.13–0.17)	0.16 (0.14–0.16)	0.17 (0.14–0.18)
Mean corpuscular volume (MCV, fL)	96.30 (88.58–108.50)	96.40 (87.55–107.50)	96.50 (88.20–105.00)	99.20 (88.32–106.00)

Notes. Continuous: median [IQR]; categorical: *n*/*N* (%). Kruskal–Wallis across age classes; exact two-sided *p*-values are reported in Section 3.1.2. Units/labels: ALT = alanine aminotransferase (U/L); AST = aspartate aminotransferase (U/L); ALP (U/L); Direct bilirubin (mg/dL); Total bilirubin (mg/dL); Albumin (g/dL); INR (unitless); Urea (mg/dL); Creatinine (mg/dL); Glucose (mg/dL); Sodium (mmol/L); Potassium (mmol/L); Total protein (g/dL); LDH/Lipase/Amylase (U/L); Hemoglobin (g/dL); Hematocrit (%); WBC (×10^9^/L); Platelets (PLT) (×10^9^/L); RDW (%); MCV (fL).

**Table 3:** Distribution of clinical scores in the whole group.

Parameter	Value	Number of Patients (*n*)	Percentage (%)
Child–Pugh	B	38	42.2%
	C	52	57.8%
Encephalopathy	Mild	31	34.44%
	Moderate	28	31.12%
	Severe	31	34.44%
Ascites	Mild	24	26.7%
	Moderate	26	28.9%
	Severe	40	44.4%

**Table 4:** Child–Pugh score distribution according to quartiles of MDA and 8-iso-PGF2α values.

Marker	Quartile	Child–Pugh		Total	*p*
		B	C		
MDA	Q1	9 (39.1%)	14 (60.9%)	23	0.331 *
	Q2	9 (40.9%)	13 (59.1%)	22	
	Q3	8 (36.4%)	14 (63.6%)	22	
	Q4	12 (52.2%)	11 (47.8%)	23	
8-iso-PGF2α	Q1	9 (39.1%)	14 (60.9%)	23	0.784 *
	Q2	9 (40.9%)	13 (59.1%)	22	
	Q3	10 (45.5%)	12 (54.5%)	22	
	Q4	10 (43.5%)	13 (56.5%)	23	

* Mann–Whitney test. Quartile cut points. MDA (µmol/L): Q1 ≤ 0.88; Q2 = (0.88–1.60]; Q3 = (1.60–3.41]; Q4 > 3.41. 8-iso-PGF2α (pg/mL): Q1 ≤ 210.14; Q2 = (210.14–246.00]; Q3 = (246.00–409.44]; Q4 > 409.44.

**Table 5:** Distribution of hepatic encephalopathy score according to quartiles of MDA and 8-iso-PGF2α values.

Marker	Quartile	Encephalopathy			Total	*p*
		Grade 1	Grade 2	Grade 3		
MDA	Q1	13 (59.1%)	6 (27.3%)	3 (13.6%)	22	0.021 *
	Q2	13 (56.5%)	6 (26.1%)	4 (17.4%)	23	
	Q3	14 (63.7%)	5 (22.7%)	3 (13.6%)	22	
	Q4	10 (45.4%)	6 (27.3%)	6 (27.3%)	22	
8-iso-PGF2α	Q1	10 (47.6%)	8 (38.1%)	3 (14.3%)	21	0.395 *
	Q2	14 (63.7%)	7 (31.8%)	1 (4.5%)	22	
	Q3	13 (59.1%)	5 (22.7%)	4 (18.2%)	22	
	Q4	10 (45.4%)	6 (27.3%)	6 (27.3%)	22	

* Kruskal–Wallis test. Quartile cut points. MDA (µmol/L): Q1 ≤ 0.88; Q2 = (0.88–1.60]; Q3 = (1.60–3.41]; Q4 > 3.41. 8-iso-PGF2α (pg/mL): Q1 ≤ 210.14; Q2 = (210.14–246.00]; Q3 = (246.00–409.44]; Q4 > 409.44.

**Table 6:** Sensitivity analysis: ascites present vs. absent by quartiles of MDA and 8-iso-PGF2α.8-iso-PGF2α.

Marker	Quartile	Ascites		Total	*p*
		Without	With		
MDA	Q1	10 (45.5%)	12 (54.5%)	22	0.453 *
	Q2	10 (43.5%)	13 (56.5%)	23	
	Q3	7 (31.8%)	15 (68.2%)	22	
	Q4	5 (22.7%)	17 (77.3%)	22	
8-iso-PGF2α	Q1	9 (42.9%)	12 (57.1%)	21	0.828 *
	Q2	8 (36.4%)	14 (63.6%)	22	
	Q3	7 (31.8%)	15 (68.2%)	22	
	Q4	6 (27.3%)	16 (72.7%)	22	

* Primary analyses use IAC three-level ascites (Low/Moderate/High = grades 1/2/3). The present table shows a binary sensitivity contrast (present vs. absent). *p*-values by Kruskal–Wallis across quartiles. Quartile cut points. MDA (µmol/L): Q1 ≤ 0.88; Q2 = (0.88–1.60]; Q3 = (1.60–3.41]; Q4 > 3.41. 8-iso-PGF2α (pg/mL): Q1 ≤ 210.14; Q2 = (210.14–246.00]; Q3 = (246.00–409.44]; Q4 > 409.44.

**Table 7:** Comparison of oxidative stress markers by Child–Pugh class (B vs. C).8-iso-PGF2α.

Marker	Quartile	Child–Pugh		Total	*p*
		Class B	Class C		
MDA	Q1	9 (39.1%)	14 (60.9%)	23	0.331 *
	Q2	9 (40.9%)	13 (59.1%)	22	
	Q3	8 (36.4%)	14 (63.6%)	22	
	Q4	12 (52.2%)	11 (47.8%)	23	
8-iso-PGF2α	Q1	9 (39.1%)	14 (60.9%)	23	<0.001 *
	Q2	9 (40.9%)	13 (59.1%)	22	
	Q3	10 (45.5%)	12 (54.5%)	22	
	Q4	10 (43.5%)	13 (56.5%)	23	

* Mann–Whitney test. Quartile cut points. MDA (µmol/L): Q1 ≤ 0.88; Q2 = (0.88–1.60]; Q3 = (1.60–3.41]; Q4 > 3.41. 8-iso-PGF2α (pg/mL): Q1 ≤ 210.14; Q2 = (210.14–246.00]; Q3 = (246.00–409.44]; Q4 > 409.44.

**Table 8:** Severe encephalopathy analyses.

Parameter	Median		*p* *
	Mild Forms	Severe Forms	
MDA	91.16	123.4	0.1162
8-iso-PGF2α	255.41	233.37	0.1998
Alb albumin	2.8	2.5	0.0001
INR	1.4	1.6	0.0022
Bilirubin (total)	2.1	5.71	<0.0001
Creatinine	1.05	1.3	0.0036
Child–Pugh (B/C distribution)	B: 38, C: 21	B: 0, C: 31	<0.0001

* Mann–Whitney test.

**Table 9:** Correlations between oxidative stress markers (MDA and 8-iso-PGF2α) and clinical and biochemical parameters.

Parameter	MDA		8-iso-PGF2α	
	r	*p*	r	*p*
Child–Pugh	−0.08	0.47	0	0.98
Encephalopathy	−0.01	0.94	0	0.96
Ascites	−0.06	0.59	−0.02	0.84
Alb albumin	−0.07	0.50	−0.05	0.62
INR	0.03	0.79	0.09	0.41
Bilirubin	−0.05	0.64	−0.05	0.60
Creatinine	0.06	0.59	0	0.97

**Table 10:** Association between composite score (age + increased MDA) and severity of liver disease.

Clinical Severity	Composite Score		*p*
	Negative	Positive	
Child–Pugh C	20 (52.6%)	32 (76.2%)	0.028
Severe hepatic encephalopathy	9 (23.7%)	21 (40.4%)	0.061

## Data Availability

The data presented in this study are available on request from the corresponding author due to anonymized data.
